# Prognostic impact of moderate secondary mitral regurgitation after optimized medical therapy in heart failure

**DOI:** 10.1093/eschf/xvag256

**Published:** 2026-10-17

**Authors:** Cristina Ferrero, Pau Codina, Josep Lupón, María Ruiz-Cueto, Elisabet Zamora, Andrea Borrellas, Cinta Llibre, Clara Badia-Molins, Andrea Carrete, Evelyn Santiago-Vacas, Mar Domingo, Javier Santesmases, Elena Ferrer-Sistach, Claudia Escabia, Julio Núñez, Stefan D Anker, Marco Metra, Javed Butler, Gregg W Stone, Victoria Delgado, Antoni Bayes-Genis

**Affiliations:** Heart Failure Clinic and Cardiology Service, University Hospital Germans Trias i Pujol, CIBERCV, Carretera del Canyet s/n, Badalona 08916, Spain; Heart Failure Clinic and Cardiology Service, University Hospital Germans Trias i Pujol, CIBERCV, Carretera del Canyet s/n, Badalona 08916, Spain; Department of Medicine, Universitat Autònoma de Barcelona, Passeig de la Vall d′Hebron 119-129, Barcelona 08035, Spain; Heart Failure Clinic and Cardiology Service, University Hospital Germans Trias i Pujol, CIBERCV, Carretera del Canyet s/n, Badalona 08916, Spain; Department of Medicine, Universitat Autònoma de Barcelona, Passeig de la Vall d′Hebron 119-129, Barcelona 08035, Spain; Department of Cardiology, Hospital Clínico Universitario de Valencia, Universitat de Valencia, Avenida Blasco Ibáñez 17, Valencia 46010, Spain; Heart Failure Clinic and Cardiology Service, University Hospital Germans Trias i Pujol, CIBERCV, Carretera del Canyet s/n, Badalona 08916, Spain; Heart Failure Clinic and Cardiology Service, University Hospital Germans Trias i Pujol, CIBERCV, Carretera del Canyet s/n, Badalona 08916, Spain; Department of Medicine, Universitat Autònoma de Barcelona, Passeig de la Vall d′Hebron 119-129, Barcelona 08035, Spain; Department of Cardiology, Hospital Clínico Universitario de Valencia, Universitat de Valencia, Avenida Blasco Ibáñez 17, Valencia 46010, Spain; Heart Failure Clinic and Cardiology Service, University Hospital Germans Trias i Pujol, CIBERCV, Carretera del Canyet s/n, Badalona 08916, Spain; Heart Failure Clinic and Cardiology Service, University Hospital Germans Trias i Pujol, CIBERCV, Carretera del Canyet s/n, Badalona 08916, Spain; Heart Failure Clinic and Cardiology Service, University Hospital Germans Trias i Pujol, CIBERCV, Carretera del Canyet s/n, Badalona 08916, Spain; Heart Failure Clinic and Cardiology Service, University Hospital Germans Trias i Pujol, CIBERCV, Carretera del Canyet s/n, Badalona 08916, Spain; Heart Failure Clinic and Cardiology Service, University Hospital Germans Trias i Pujol, CIBERCV, Carretera del Canyet s/n, Badalona 08916, Spain; Department of Cardiology, Hospital Clínico Universitario de Valencia, Universitat de Valencia, Avenida Blasco Ibáñez 17, Valencia 46010, Spain; Heart Failure Clinic and Cardiology Service, University Hospital Germans Trias i Pujol, CIBERCV, Carretera del Canyet s/n, Badalona 08916, Spain; Heart Failure Clinic and Cardiology Service, University Hospital Germans Trias i Pujol, CIBERCV, Carretera del Canyet s/n, Badalona 08916, Spain; Department of Medicine, Universitat Autònoma de Barcelona, Passeig de la Vall d′Hebron 119-129, Barcelona 08035, Spain; Heart Failure Clinic and Cardiology Service, University Hospital Germans Trias i Pujol, CIBERCV, Carretera del Canyet s/n, Badalona 08916, Spain; Department of Medicine, Universitat Autònoma de Barcelona, Passeig de la Vall d′Hebron 119-129, Barcelona 08035, Spain; Heart Failure Clinic and Cardiology Service, University Hospital Germans Trias i Pujol, CIBERCV, Carretera del Canyet s/n, Badalona 08916, Spain; Department of Cardiology, Hospital Clínico Universitario de Valencia, Universitat de Valencia, Avenida Blasco Ibáñez 17, Valencia 46010, Spain; Department of Cardiology (CVK) of German Heart Center Charité, German Centre for Cardiovascular Research (DZHK) Partner Site Berlin, Charité Universitätsmedizin, Berlin, Germany; Department of Medical and Surgical Specialties, Radiological Sciences and Public Health, Institute of Cardiology, University of Brescia, Spedali Civili di Brescia, Brescia, Italy; Baylor Scott and White Research Institute, Dallas, TX, USA; Division of cardiology, Department of Medicine, Icahn School of Medicine at Mount Sinai, New York, NY, USA; Heart Failure Clinic and Cardiology Service, University Hospital Germans Trias i Pujol, CIBERCV, Carretera del Canyet s/n, Badalona 08916, Spain; Heart Failure Clinic and Cardiology Service, University Hospital Germans Trias i Pujol, CIBERCV, Carretera del Canyet s/n, Badalona 08916, Spain; Department of Medicine, Universitat Autònoma de Barcelona, Passeig de la Vall d′Hebron 119-129, Barcelona 08035, Spain; Department of Cardiology, Hospital Clínico Universitario de Valencia, Universitat de Valencia, Avenida Blasco Ibáñez 17, Valencia 46010, Spain

**Keywords:** Heart failure, Secondary mitral regurgitation, Prognosis

## Abstract

**Background and Aims:**

The prognostic significance of moderate secondary mitral regurgitation (MR) after optimized guideline-directed medical therapy (GDMT) in heart failure (HF) remains uncertain.

**Methods:**

We analysed 2468 ambulatory HF patients with paired transthoracic echocardiography at baseline and after 12 months of optimized GDMT. The primary composite was all-cause death or first heart failure hospitalization (HFH). Secondary endpoints included all-cause mortality and recurrent HFHs.

**Results:**

The mean age of patients was 66.2 ± 12.5 years, 70.6% were male, and the mean baseline left ventricular ejection fraction was 36.1% ± 13.6%. At 12 months, 80.5% of patients had no/mild MR, 15.6% had moderate MR, and 3.9% had severe MR. During a median 6.9-year follow-up, 1240 patients experienced the primary endpoint. Compared with no/mild MR, both moderate and severe MR at 12 months were independently associated with a higher risk of the primary composite endpoint [moderate: hazard ratio (HR) 1.69, 95% confidence interval (CI) 1.47–1.95; severe: HR 1.81, 95% CI 1.44–2.29; both *P* < .001). Moderate MR was also independently associated with increased all-cause mortality (HR 1.54, 95% CI 1.32–1.79; *P* < .001) and recurrent HFHs (incidence rate ratio 2.17, 95% CI 1.81–2.60). Heart failure hospitalization rates were 9.03, 20.0, and 26.1 per 100 person-years in patients with no/mild, moderate, and severe MR, respectively. Among patients with moderate MR at 12 months, outcomes did not differ according to baseline MR severity.

**Conclusions:**

In this large-scale study, residual moderate secondary MR after 12 months of optimized GDMT was common and was independently associated with increased long-term mortality and HFHs.

## Graphical Abstract

**Graphical Abstract:**
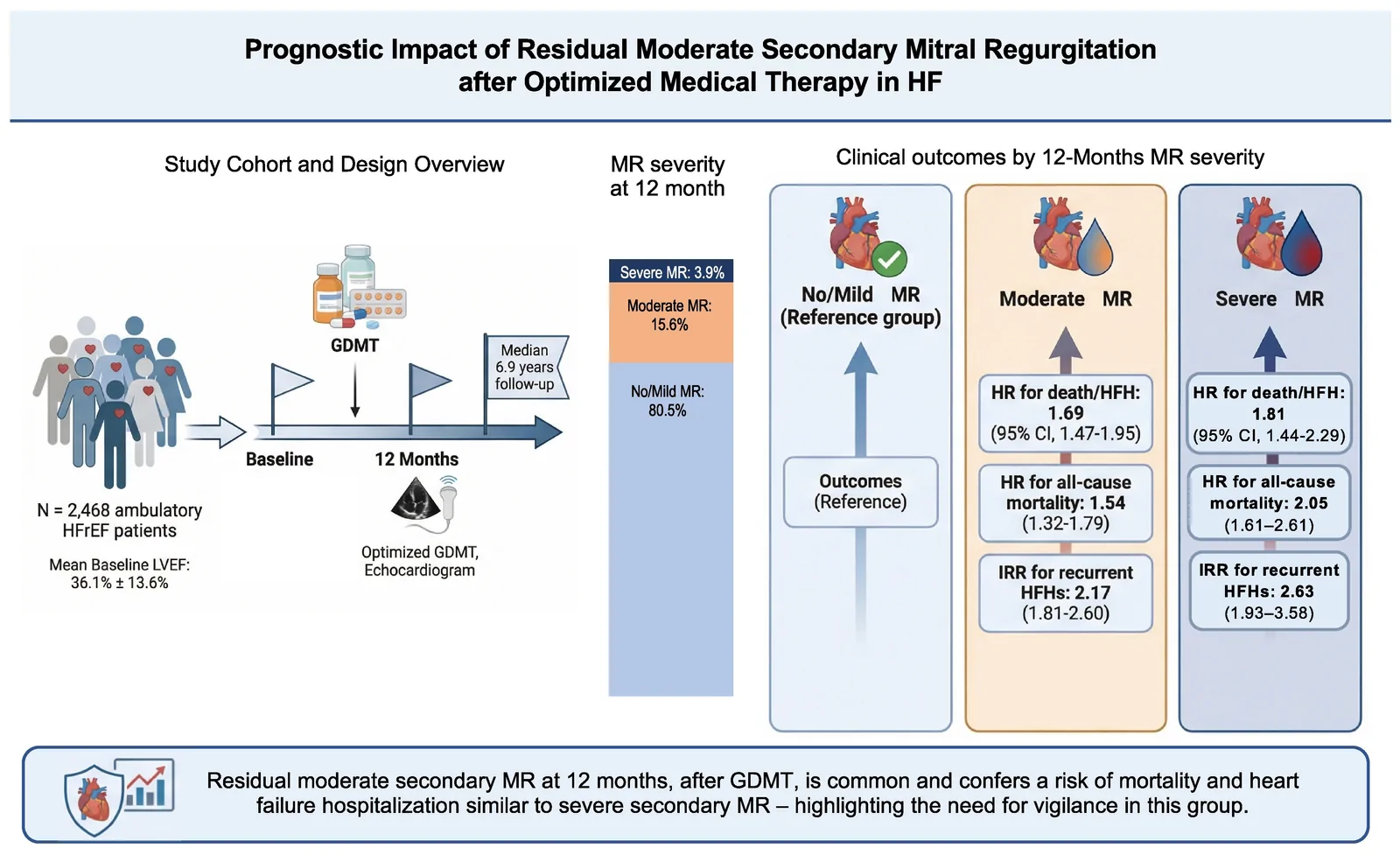
Prognostic impact of residual moderate secondary MR after optimized medical therapy in HF.

## Introduction

Secondary mitral regurgitation (MR) is a common consequence of left ventricular remodelling in patients with heart failure with reduced ejection fraction (HFrEF), and its occurrence is independently associated with increased mortality and heart failure hospitalizations (HFHs).^1,2^ Although major advances in guideline-directed medical therapy (GDMT) and device-based therapies have improved outcomes in HFrEF,^3^ residual secondary MR after GDMT optimization remains frequent and continues to confer excess risk.^4,5^ Importantly, secondary MR is a dynamic condition. In a large real-world cohort of ambulatory HFrEF patients undergoing systematic reassessment after GDMT optimization, more than half improved from significant to non-significant MR within 12 months, whereas persistent significant MR was associated with worse long-term outcomes.^6^

Mitral transcatheter edge-to-edge repair (M-TEER) has emerged as an effective therapeutic option for selected patients with severe secondary MR, and landmark trials have demonstrated reductions in HF events and improved quality of life.^7,8^ Consequently, severe secondary MR is now recognized as a target for intervention in contemporary guidelines.^3^ However, the clinical relevance of moderate secondary MR remains uncertain. Observational studies suggest that moderate secondary MR is associated with intermediate outcomes between mild and severe disease.^9,10^ In addition, registry data suggest that M-TEER may improve symptomatic status in selected patients with moderate secondary MR.^11^ Nevertheless, these single-arm studies lacked an untreated control group and did not study the long-term natural history of moderate MR in patients receiving contemporary optimized GDMT. Interpretation is further complicated by evolving echocardiographic grading criteria and the multiparametric nature of secondary MR assessment, which may influence classification and prognostic interpretation.^10^ As a result, the prognostic significance of residual moderate secondary MR after GDMT optimization, and the clinical characteristics of patients in whom it persists, remain incompletely characterized.

To address these gaps, we performed a long-term analysis of ambulatory HF patients enrolled in a structured multidisciplinary HF program with systematic echocardiographic reassessment after 12 months of optimized GDMT. The primary aim was to evaluate the prognostic impact of secondary MR severity at follow-up, with particular focus on patients with persistent moderate secondary MR after optimized GDMT.

## Methods

### Study population

All consecutive ambulatory patients referred to the multidisciplinary HF Unit at Germans Trias i Pujol University Hospital, Badalona, Spain, between August 2001 and December 2023, were considered for inclusion. During this 22 year period, both the clinical pathways and the referral area—covering approximately 850 000 inhabitants in the northern Barcelona metropolitan region—remained stable.

The criteria for referral to the HF clinic were HF according to the European Society of Cardiology definition, with at least one hospitalization and/or reduced systolic function. Patients were managed within a standardized, protocol-driven programme to optimize GDMT and device therapy when indicated.^6^

The present analysis included all patients with paired transthoracic echocardiograms (TTEs) available at baseline and at 12 months. Patients were excluded if they had undergone surgical mitral valve replacement (mechanical or bioprosthetic) or M-TEER before baseline or at any time during follow-up. Patients with predominant primary (degenerative) MR were also excluded. Patients who died within the first 12 months were excluded to allow classification according to secondary MR severity at follow-up.

Follow-up echocardiograms were completed by 31 December 2024, and clinical outcomes were updated until 30 June 2025, ensuring a minimum follow-up of 6 months after the 12 month echocardiogram. Maximum follow-up was limited to 10 years.

The study was conducted in accordance with the Declaration of Helsinki and approved by the local ethics committee (code REGI-UNIC PI-18-037). All patients provided written informed consent for the use of their clinical and imaging data for research purposes.

### Echocardiography

Comprehensive TTE was performed at baseline and at 12 months following standardized institutional protocols. Studies were acquired with the patient in left lateral decubitus position, using parasternal long- and short-axis and apical two-, three-, and four-chamber views.

Secondary MR severity was graded according to contemporary guideline recommendations, using a multiparametric, integrated approach that included qualitative, semiquantitative and quantitative parameters, as previously described.^6^ Mitral regurgitation was categorized as none/mild, moderate, or severe. Quantitative thresholds were applied when available: moderate MR was defined by an effective regurgitant orifice area (EROA) of 20–29 mm^2^ or a regurgitant volume (RVol) of 30–44 ml, and severe MR by an EROA ≥40 mm^2^ (or ≥30 mm^2^ in the presence of an elliptical orifice) or RVol ≥45 ml. Complementary indicators of severity included vena contracta width, pulmonary venous flow reversal, and jet area. All echocardiographic assessments were performed by experienced cardiologists with expertise in valvular imaging, within the same core laboratory.^6^

### Outcomes

Patients were followed from the date of the 12 month echocardiogram until 30 June 2025, or death, whichever occurred first, with a maximum follow-up of 10 years. The primary endpoint was the composite of all-cause death or first HFH. Secondary endpoints included all-cause mortality and first and recurrent HFHs. Events were prospectively recorded and validated through hospital electronic medical records and regional health databases.

### Statistical analysis

Continuous variables are expressed as mean ± SD or median [interquartile range], as appropriate. Categorical variables are presented as absolute numbers and percentages. Normality was assessed using Q–Q plots. Patients were classified according to MR severity at 12 months into three groups: none/mild, moderate and severe MR. Global comparisons were performed using one-way ANOVA or Kruskal–Wallis tests for continuous variables and Pearson χ^2^ tests for categorical variables. For comparisons across the three MR groups, post-hoc pairwise analyses were performed when appropriate. Scheffé tests were used after one-way ANOVA, Bonferroni-adjusted pairwise comparisons after Kruskal–Wallis tests, and Bonferroni-adjusted χ^2^ or Fisher’s exact tests for categorical variables, as appropriate.

Time-to-event analyses for the composite endpoint of all-cause mortality or first HFH were performed using Cox proportional hazards regression. Univariable and multivariable models were fitted. The proportional hazards assumption was formally assessed using Schoenfeld residuals for each covariate and globally for all Cox models. Multivariable models were adjusted for age, sex, ischaemic vs non-ischaemic aetiology, left ventricular ejection fraction (LVEF), HF duration, diabetes mellitus, and MR severity group. Covariates were selected based on their association with the outcome in univariable analyses and/or their established clinical relevance (sex). No automated variable-selection procedure was used. Time to all-cause mortality was analysed using Cox proportional hazards models following the same approach. For time to first HFH, competing risk analyses were conducted using the Fine and Gray subdistribution hazard models, with death from any cause treated as a competing event. Patients without HFH or death were censored at the end of follow-up. As a sensitivity analysis, the multivariable Cox models were additionally adjusted for calendar year of enrolment to account for temporal changes in HF management.

Crude incidence rates of all HF-related hospitalizations (first and recurrent) were calculated for each MR group and expressed as admissions per 100 person-years. Time spent in hospital was subtracted from the total follow-up time to account for periods not at risk. Pairwise crude comparisons of hospitalization rates between MR groups were performed using negative binomial regression with Holm-adjusted *P*-values. Recurrent HFHs were further analysed using multivariable negative binomial regression models with log-transformed person-time as an offset. Models were adjusted for the same covariates, and results are presented as incidence rate ratios (IRRs) with 95% confidence interval (CI). In that multivariable recurrent event models, out-of-hospital death due to HF was considered a terminal event.

Cumulative incidence curves were generated using the Kaplan–Meier estimates for the primary end-point and all-cause death, and the Fine-Gray model for first HFH. A predefined subanalysis was conducted in patients with moderate secondary MR at 12 months, in whom outcomes were evaluated according to baseline MR severity (none/mild, moderate, or severe) using multivariable Cox proportional hazards models adjusted for the same covariates. In addition, a separate subanalysis was performed to assess the prognostic impact of heart failure with improved ejection fraction (HFimpEF) within this subgroup. All statistical tests were two-sided, and a *P*-value <.05 was considered statistically significant. Statistical analyses were performed using IBM SPSS Statistics, version 25 (IBM Corp., Armonk, NY, USA) and R version 4.5.2 (R Foundation for Statistical Computing, Vienna, Austria).

## Results

Between August 2001 and December 2024, 3634 consecutive patients were referred to the multidisciplinary HF clinic. Of these, 186 patients were excluded due to a prior history of mitral valve intervention (95 mechanical valve replacement, 44 bioprosthetic valve replacement, and 47M-TEER). An additional five patients who underwent heart transplantation within the first year of follow-up and 335 patients who died during that first year were excluded. Furthermore, 640 patients were excluded because of missing MR grading at baseline and/or at the 12 month follow-up (Supplementary Table S1). The final study population thus comprised 2468 patients with paired baseline and 12 month echocardiographic assessments (Supplementary Figure S1).

Characteristics of the study population are summarized in *Table 1*, and medical and device therapies at baseline and 12 months are detailed in *Table 2*.

**Table 1:** Clinical and echocardiographic characteristics according to secondary MR severity at 12 months

	Overall cohort (*n* = 2468)	None/mild MR^a^ (*n* = 1986)	Moderate MR^b^ (*n* = 386)	Severe MR^c^ (*n* = 96)	Global *P*-value	Pairwise adjusted *P*-values		
						1 vs 2	1 vs 3	2 vs 3
Baseline clinical characteristics								
Age (years)	66.2 ± 12.5	65.2 ± 12.6	69.6 ± 11.8	72.5 ± 10.8	<.001	<.001	<.001	.131
Male sex, *n* (%)	1743 (70.6%)	1440 (72.5%)	246 (63.7%)	57 (59.4%)	<.001	.003	.015	1.000
HF duration (months)	6 [1–36]	6 [1–34]	9 [2–48]	14 [3–60]	<.001	.003	.002	.351
Ischaemic aetiology, *n* (%)	1100 (44.6%)	879 (44.3%)	171 (44.3%)	50 (52.1%)	.319	1.000	.396	.513
NYHA class, *n* (%)					<.001	<.003	<.003	.012
I	215 (8.7%)	195 (9.8%)	19 (4.9%)	1 (1.0%)				
II	1758 (71.3%)	1424 (71.8%)	273 (70.7%)	61 (63.5%)				
III	485 (19.7%)	359 (18.1%)	94 (24.4%)	32 (33.3%)				
IV	7 (0.3%)	5 (0.3%)	0 (0.0%)	2 (2.1%)				
Atrial fib/flutter, *n* (%)	525 (21.3%)	362 (18.2%)	129 (33.4%)	34 (35.4%)	<.001	<.003	<.003	1.000
Diabetes mellitus, *n* (%)	993 (40.2%)	797 (40.1%)	163 (42.2%)	33 (34.4%)	.365	1.000	.783	.483
eGFR (mL/min/1.73 m^2^)	66.6 ± 26.7	68.2 ± 26.6	61.0 ± 26.6	55.4 ± 23.7	<.001	<.001	<.001	.382
Haemoglobin (g/dL)	13.2 ± 1.8	13.3 ± 1.8	12.8 ± 1.8	12.6 ± 2.0	<.001	<.001	.006	.706
NT-proBNP (pg/mL)	1460 [613–3345]	1278 [532–3070]	2129 [1121–4509]	2411 [1258–6992]	<.001	<.001	<.001	.849
Baseline echocardiography								
LVEF (%)	36.1 ± 13.6	35.9 ± 13.2	37.3 ± 15.4	35.2 ± 15.5	.14	.167	.910	.418
LVEDD (mm)	59.0 ± 9.3	58.8 ± 9.1	58.9 ± 9.7	62.3 ± 11.4	.002	.986	.002	.006
LA diameter (mm)	46.7 ± 7.8	46.0 ± 7.6	49.4 ± 7.6	51.3 ± 9.8	<.001	<.001	<.001	.111
sPAP (mmHg)	44.0 ± 13.0	42.9 ± 12.5	46.6 ± 13.6	51.3 ± 14.8	<.001	<.001	<.001	.022
Echocardiography at 12 months								
LVEF (%)	44.5 ± 13.7	45.5 ± 13.1	41.3 ± 15.3	38.4 ± 16.5	<.001	<.001	<.001	.189
LVEDD (mm)	55.6 ± 9.6	54.8 ± 9.1	58.0 ± 10.2	61.4 ± 11.8	<.001	<.001	<.001	.008
LA diameter (mm)	46.0 ± 8.1	45.0 ± 7.7	49.5 ± 7.9	53.7 ± 8.7	<.001	<.001	<.001	<.001
sPAP (mmHg)	40.6 ± 12.8	38.3 ± 11.4	45.3 ± 13.3	52.7 ± 15.4	<.001	<.001	<.001	<.001

Values are presented as mean ± standard deviation, *n* (%), or median [interquartile range]. Pairwise columns compare none/mild^a^ vs moderate^b^, none/mild vs severe^c^, and moderate vs severe MR, respectively.

Atrial fib/flutter, atrial fibrillation/flutter; eGFR, estimated glomerular filtration rate; HF, heart failure; LA, left atrium; LVEDD, left ventricular end-diastolic diameter; LVEF, left ventricular ejection fraction; NT-proBNP, N-terminal pro–B-type natriuretic peptide; NYHA, New York Heart Association; sPAP, systolic pulmonary artery pressure; MR, mitral regurgitation.

**Table 2:** Medical and device therapy at baseline and 12 months according to secondary MR severity at 12 months

	Overall cohort (*n* = 2468)	None/mild MR^a^ (*n* = 1986)	Moderate MR^b^ (*n* = 386)	Severe MR^c^ (*n* = 96)	Global *P*-value	Pairwise adjusted *P*-values		
						1 vs 2	1 vs 3	2 vs 3
Baseline treatment								
ACEi/ARB/ARNI, *n* (%)	1885 (76.4%)	1528 (76.9%)	284 (73.6%)	73 (76.0%)	.284	1.000	1.000	1.000
β-Blocker, *n* (%)	1920 (77.8%)	1552 (78.1%)	303 (78.5%)	65 (67.7%)	.052	0.078	0.078	0.078
MRA, *n* (%)	1148 (46.5%)	939 (47.3%)	169 (43.8%)	40 (41.7%)	.282	1.000	1.000	1.000
SGLT2i, n (%)	258 (10.5%)	228 (11.5%)	29 (7.5%)	1 (1.0%)	<.001	0.057	0.057	0.057
Loop diuretic, *n* (%)	1786 (72.4%)	1381 (69.5%)	325 (84.2%)	80 (83.3%)	<.001	1.000	1.000	1.000
Digoxin, *n* (%)	392 (15.9%)	276 (13.9%)	90 (23.3%)	26 (27.1%)	<.001	1.000	1.000	1.000
ICD, *n* (%)	208 (8.4%)	168 (8.5%)	32 (8.3%)	8 (8.3%)	.993	1.000	1.000	1.000
CRT, *n* (%)	97 (3.9%)	74 (3.7%)	17 (4.4%)	6 (6.2%)	.403	1.000	1.000	1.000
Treatment at 12 months								
ACEi/ARB/ARNI, *n* (%)	2158 (87.4%)	1757 (88.5%)	326 (84.5%)	75 (78.1%)	.002	0.081	0.006	0.414
β-Blocker, *n* (%)	2248 (91.1%)	1823 (91.8%)	346 (89.6%)	79 (82.3%)	.003	0.498	0.003	0.138
MRA, *n* (%)	1584 (64.2%)	1288 (64.9%)	238 (61.7%)	58 (60.4%)	.358	0.690	1.000	1.000
SGLT2i, *n* (%)	537 (21.8%)	468 (23.6%)	64 (16.6%)	5 (5.2%)	<.001	0.009	<0.003	0.012
Loop diuretic, *n* (%)	2058 (83.4%)	1613 (81.2%)	353 (91.5%)	92 (95.8%)	<.001	<0.003	<0.003	0.447
Digoxin, *n* (%)	545 (22.1%)	386 (19.4%)	120 (31.1%)	39 (40.6%)	<.001	<0.003	<0.003	0.225
ICD, *n* (%)	291 (11.8%)	235 (11.8%)	46 (11.9%)	10 (10.4%)	.912	1.000	1.000	1.000
CRT, *n* (%)	163 (6.6%)	129 (6.5%)	24 (6.2%)	10 (10.4%)	.302	1.000	0.399	0.450

Values are presented as *n* (%). Pairwise columns compare none/mild^a^ vs moderate^b^, none/mild vs severe^c^, and moderate vs severe MR, respectively.

ACEi, angiotensin-converting enzyme inhibitor; ARB, angiotensin II receptor blocker; ARNI, angiotensin receptor–neprilysin inhibitor; CRT, cardiac resynchronization therapy; ICD, implantable cardioverter-defibrillator; MRA, mineralocorticoid receptor antagonist; SGLT2i, sodium–glucose cotransporter 2 inhibitor; MR, mitral regurgitation.

The mean age was 66.2 ± 12.5 years, and 1743 patients (70.6%) were male. Diabetes mellitus was present in 993 patients (40.2%), and the median duration of HF was 6.0 [1.0–36.0] months. The median baseline N-terminal pro–B-type natriuretic peptide (NT-proBNP) was 1460 [613–3345] pg/ml, and most patients were in New York Heart Association functional class II (71.3%) at study entry.

Baseline mean LVEF was 36.1% ± 13.6%, and 1796 patients (72.8%) had HFrEF (LVEF ≤40%). After 12 months of optimized GDMT, the mean LVEF increased to 44.5% ± 13.7% (*P* < .001).

The distribution of secondary MR severity also changed during follow-up. At baseline, MR was absent or mild in 1683 patients (68.2%), moderate in 613 (24.8%), and severe in 172 (7.0%). At 12 month follow-up, 1986 patients (80.5%) were classified as having no or mild MR, 386 (15.6%) as moderate MR, and 96 (3.9%) as severe MR (*P* < .001 compared with baseline) (Supplementary Figure S2).

Patients with moderate or severe secondary MR at 12 months exhibited a more advanced HF phenotype, characterized by older age, worse functional status, higher prevalence of atrial fibrillation, greater cardiac remodelling, impaired renal function, higher NT-proBNP levels, and a poorer structural and functional response to optimized medical therapy compared with patients with no or mild MR.

### Evolution of moderate secondary MR

Among patients with moderate secondary MR at 12 months (*n* = 386), this phenotype resulted from three distinct baseline trajectories. Moderate MR persisted from baseline in 195 patients (50.5%), 146 patients (37.8%) progressed from no or mild MR, and 45 patients (11.7%) improved from severe MR (Supplementary Figure S3).

### Clinical outcomes

During a median follow-up of 6.9 years (IQR 4.4–8.4) after the 12-month echocardiogram, 1240 patients experienced the primary composite endpoint of all-cause mortality or first HFH. Overall, 1037 patients died, 635 experienced at least one HFH, and a total of 1318 HFHs were recorded in these 635 patients (mean 2.08 ± 1.67 HFHs per patient).

A strong association was present between residual secondary MR severity at 12 months and the risk of the primary composite endpoint (*Figure 1A*). Patients with moderate or severe secondary MR had a higher incidence of the composite outcome compared with those with mild or no MR, whereas no significant differences were observed between moderate and severe MR. After multivariable adjustment, patients with moderate secondary MR had a 69% higher risk of the composite endpoint (HR 1.69, 95% CI 1.47–1.95), while those with severe secondary MR had an 81% higher risk (HR 1.81, 95% CI 1.44–2.29) compared with patients with mild or no MR (*Table 3*).

**Figure 1:**
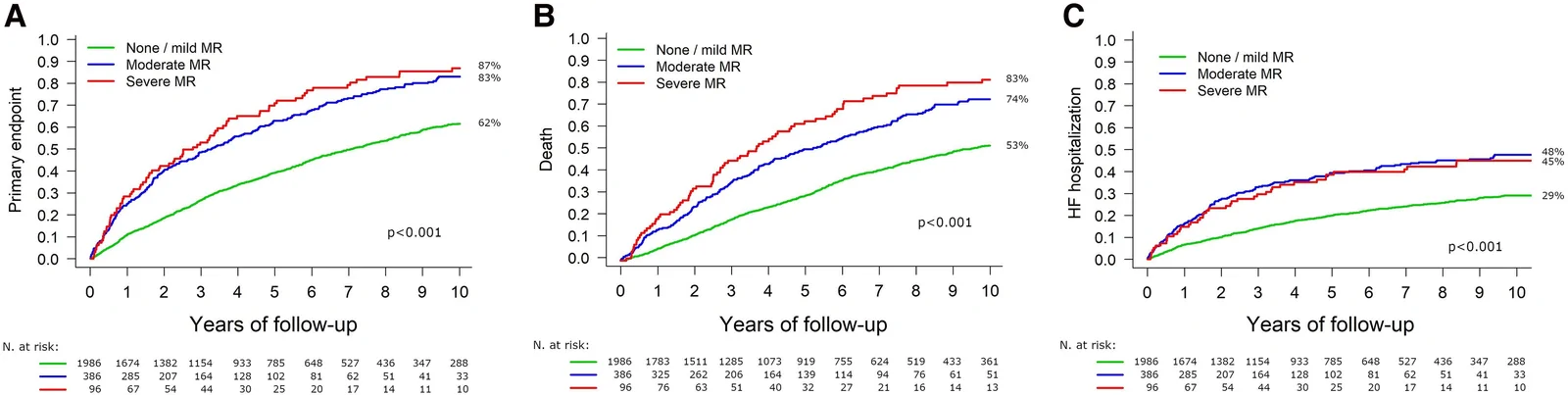
Clinical outcomes according to secondary mitral regurgitation (MR) severity at 12 months.

**Table 3:** Multivariable predictors of time to first death or heart failure hospitalization

Variable	Adjusted HR (95% CI)	*P* value
Secondary MR group 2 vs 1	1.691 (1.469–1.945)	<.001
Secondary MR group 3 vs 1	1.813 (1.435–2.290)	<.001
Age (per 5 years)	1.269 (1.234–1.306)	<.001
Female sex	0.860 (0.755–0.980)	.024
Ischaemic aetiology	1.188 (1.053–1.341)	.005
LVEF at 12 months (per 5%)	0.950 (0.928–0.973)	<.001
HF duration (log)	1.065 (1.032–1.098)	<.001
Diabetes mellitus	1.485 (1.326–1.663)	<.001

MR group 1 corresponds to mild or no MR, group 2 to moderate MR, and group 3 to severe MR.

HF, heart failure; IRR, incidence rate ratio; LVEF, left ventricular ejection fraction; MR, mitral regurgitation.

Residual secondary MR severity at 12 months was independently associated with all-cause mortality (*Figure 1B*). By multivariable analysis, moderate secondary MR was associated with a 54% higher mortality risk (hazard ratio [HR] 1.54, 95% CI 1.32–1.79), while severe secondary MR conferred an approximately two-fold increase in mortality risk (HR 2.05, 95% CI 1.61–2.61) compared with no/mild MR (*Table 4*).

**Table 4:** Multivariable predictors of all-cause mortality

Variable	Adjusted HR (95% CI)	*P* value
Secondary MR group 2 vs 1	1.537 (1.320–1.789)	<.001
Secondary MR group 3 vs 1	2.049 (1.609–2.609)	<.001
Age (per 5 years)	1.370 (1.326–1.416)	<.001
Female sex	0.771 (0.668–0.890)	<.001
Ischaemic aetiology	1.206 (1.056–1.378)	.006
LVEF at 12 months (per 5%)	0.961 (0.937–0.986)	.002
HF duration (log)	1.062 (1.027–1.100)	.001
Diabetes mellitus	1.544 (1.365–1.747)	<.001

MR group 1 corresponds to mild or no MR, group 2 to moderate MR, and group 3 to severe MR.

HF, heart failure; LVEF, left ventricular ejection fraction; IRR, incidence rate ratio. MR, mitral regurgitation.

In the sensitivity analysis, additional adjustment for calendar year of enrolment did not materially alter the association between MR severity and the primary composite endpoint or all-cause mortality. Calendar year of enrolment was not independently associated with either the primary composite endpoint (HR 0.99, 95% CI 0.98–1.002; *P* = .11) or all-cause mortality (HR 0.99, 95% CI 0.98–1.004; *P* = .24), and similar findings were observed in the predefined subgroup analyses.

The risk of a first HFH was also higher in patients with moderate and severe secondary MR (*Figure 1C*). After multivariable adjustment and accounting for the competing risk of death, moderate secondary MR was strongly associated with first HFH [subdistribution HR (sHR) 1.79, 95% CI 1.47–2.17], as much or more so than severe secondary MR (sHR 1.48, 95% CI 1.05–2.10), compared with patients with mild or no MR (Supplementary Table S2).

Crude rates of all (first and recurrent) HFHs increased stepwise with MR severity, from 9.03 admissions per 100 person-years (95% CI 8.45–9.64) in patients with no or mild MR to 20.0 admissions per 100 person-years (95% CI 17.9–22.3) in those with moderate MR to 26.1 admissions per 100 person-years (95% CI 21.3–31.7) in those with severe MR (*Figure 2*). Pairwise crude comparisons showed higher rates in both moderate and severe MR compared with no/mild MR, whereas no significant difference was observed between moderate and severe MR.

**Figure 2:**
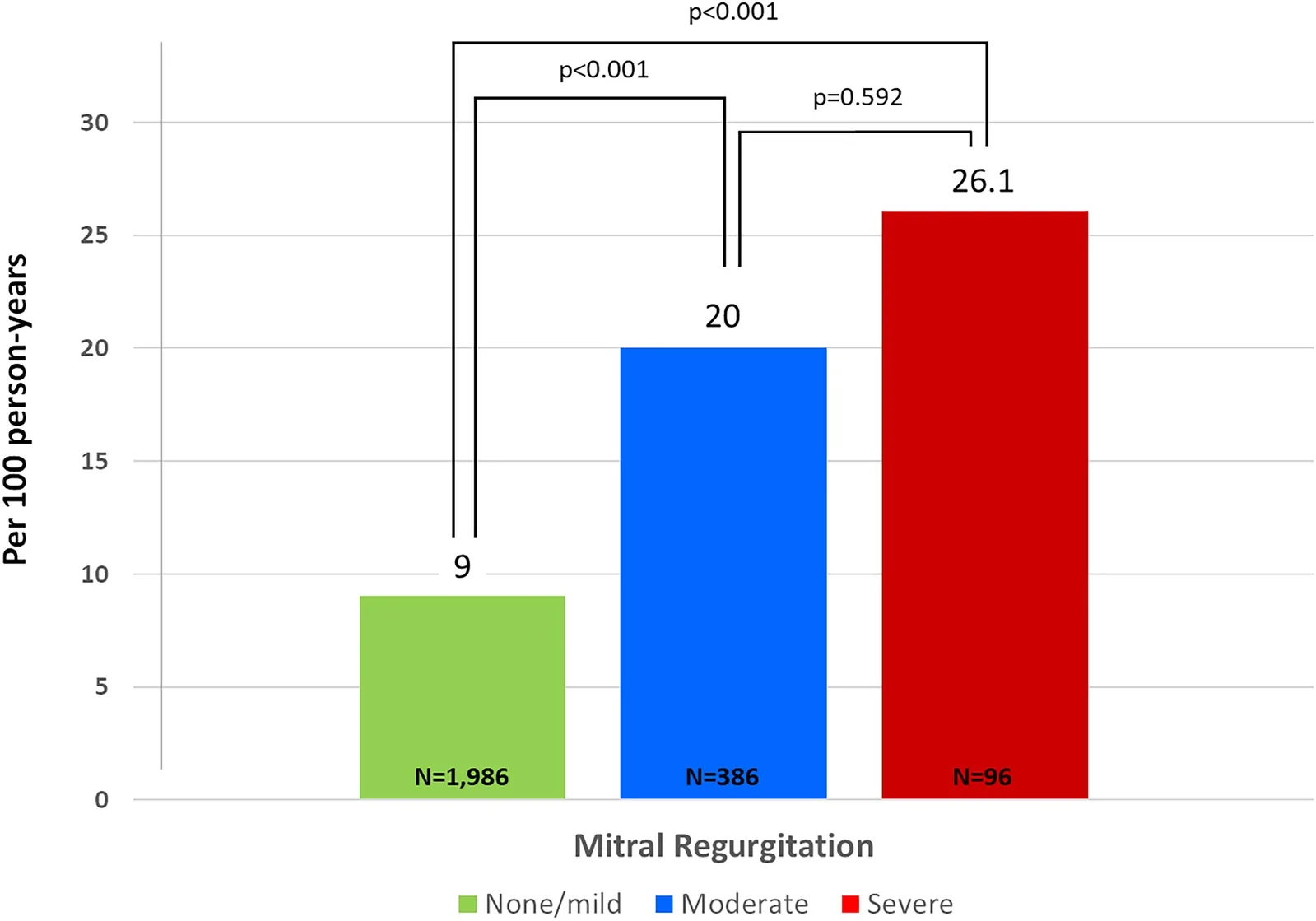
Recurrent heart failure hospitalizations according to secondary mitral regurgitation (MR) severity at 12 months.

In multivariable negative binomial regression analyses accounting for person-time at risk, both moderate and severe secondary MR were independently associated with all HFHs. Compared with no/mild MR, moderate MR was associated with a 2.17-fold increase in HFH (IRR 2.17, 95% CI 1.81–2.60), while severe MR conferred a 2.63-fold increase in HFH (IRR 2.63, 95% CI 1.93–3.58) (Supplementary Table S3).

A predefined subanalysis was conducted in patients with moderate secondary MR at 12 months, in whom clinical outcomes were evaluated according to baseline MR severity (none/mild, moderate or severe). Among patients with moderate secondary MR at 12 months, clinical outcomes did not differ according to baseline MR severity. Baseline MR severity was not associated with the primary composite endpoint, all-cause mortality, first HFH, or all HFHs in univariable or multivariable analyses. Overall, outcomes were comparable across all baseline MR trajectories once moderate secondary MR persisted at 12 months (Supplementary Figures S4–5 and Supplementary Tables S4–5).

Among patients with moderate secondary MR at 12 months, 61 (15.8%) met criteria for HFimpEF, defined as an absolute increase in LVEF >10 percentage points from baseline and an LVEF >40% at 12 months. In the exploratory multivariable Cox regression analysis, HFimpEF was associated with an HR of 0.83 (95% CI 0.55–1.25); *P* = .371. The small sample size and wide CI preclude firm conclusions, and this finding should therefore be considered hypothesis-generating (*Graphical abstract*).

## Discussion

This study provides novel insights into the prognostic significance of secondary MR in patients with HF (most of whom had HFrEF) managed within a structured, protocol-driven programme of GDMT. Our findings demonstrate a clear and graded association between MR severity at 12 months (despite optimized GDMT) and adverse long-term clinical outcomes. Specifically, after adjustment for meaningful covariates, patients with moderate or severe residual secondary MR at follow-up experienced substantially higher risks of all-cause mortality, first HFH, recurrent HFHs, and the composite endpoint when compared with those presenting with no or mild MR. These results underscore that residual moderate secondary MR persisting after GDMT optimization carries meaningful prognostic implications and may not be a benign biomarker.

The prevalence of moderate secondary MR in HFrEF populations has been reported with considerable variability across studies, ranging from as low as 6% to as high as 33%.^2,12–16^ This heterogeneity likely reflects important differences in patient selection, HF severity, echocardiographic methodology, and intensity of medical therapy, as well as MR definitions and grading strategies. In this context, our data provide a contemporary estimate of moderate secondary MR burden in a well-characterized HF cohort treated under systematic GDMT optimization. In a large recent registry reported by Bartko et al., moderate secondary MR was observed in approximately 44% of patients with HFrEF.^10^ In contrast, 25% of patients in our prospective cohort had moderate secondary MR at baseline and 16% of patients had moderate secondary MR after 12 months of optimized GDMT, reinforcing that moderate MR remains a frequent clinical entity even under contemporary and standardized care.

The adverse causative prognostic impact of severe secondary MR has been firmly established by randomized trials that demonstrated that eliminating this lesion improves clinical outcomes.^7,8,17^ Pagnesi et al. reported an 85% higher risk of the composite of all-cause death or HFH in patients with moderate-to-severe MR after GDMT optimization (adjusted HR 1.85; 95% CI 1.43–2.39; *P* < .001).^18^ Nevertheless, the clinical relevance of moderate secondary MR itself has remained less clearly defined, particularly in the setting of optimized GDMT. Several observational studies have suggested a continuous relationship between the severity of secondary MR and clinical outcomes. Benfari et al. demonstrated a progressive increase in mortality with increasing EROA, even across moderate categories of MR severity.^9^ Similarly, Sarrazyn et al. reported high rates of mortality and HFH among patients with moderate secondary MR in the context of advanced ventricular dysfunction.^19^ However, many of these prior analyses were derived from heterogeneous, non-protocolized cohorts with inconsistent medical therapy and without systematic reassessment of MR severity over time. As a result, it has remained uncertain whether moderate secondary MR retains prognostic relevance after GDMT optimization or represents a transient stage that frequently resolves with HF therapy.

In line with previous studies,^17,18,20^ our group previously reported that secondary MR is a highly dynamic condition that may regress in a substantial proportion of HFrEF patients following GDMT,^6^ a finding with important prognostic implications. The present study extends these observations by specifically focusing on patients with moderate secondary MR persisting after GDMT optimization. Importantly, we demonstrate that residual moderate secondary MR at 12 months is independently associated with increased risks of mortality and HFHs, even after adjustment for relevant clinical and echocardiographic covariates. These findings clarify that moderate secondary MR is a clinically meaningful marker of residual risk.

Among patients with moderate secondary MR at follow-up, baseline MR severity did not influence subsequent outcomes. This suggests that prognosis is primarily driven by the severity of residual MR after treatment optimization rather than by the initial degree of MR at presentation. This supports a dynamic, treatment-responsive framework for secondary MR assessment, in which systematic echocardiographic reassessment after GDMT optimization provides essential prognostic information beyond baseline evaluation and allows identification of patients who remain at high risk despite best medical management.

From a mechanistic standpoint, persistent moderate secondary MR likely reflects sustained distortion of mitral valve geometry that may lead to progressive adverse left ventricular remodelling. These structural changes contribute to chronic volume overload, elevated left ventricular filling pressures and progressive HF decompensation.^21^ Moderate secondary MR may therefore represent not only a marker of disease severity but also a mediator of hemodynamic deterioration through increased volume overload, reduced forward stroke volume, and amplification of ventricular stress. In this context, residual moderate secondary MR may identify patients in whom GDMT has been insufficient to halt the vicious cycle of ventricular remodelling and regurgitation progression. Systematic follow-up echocardiography should therefore be considered an integral component of secondary MR management to distinguish patients with treatment-responsive regurgitation from those with persistent residual disease associated with higher risk.

As the clinical understanding of moderate secondary MR continues to evolve, so does the debate regarding optimal management strategies. Guideline-directed medical therapy remains the cornerstone of treatment in HFrEF, as it can promote reverse remodeling^22^ and reduce MR severity in a substantial proportion of patients.^3,17,18,20^ Indeed, Kresoja et al. showed that greater GDMT intensity was associated with lower all-cause mortality in patients with HFrEF and secondary MR undergoing M-TEER.^23^ However, our findings suggest that patients with persistent moderate secondary MR despite optimized therapy represent a subgroup with residual risk who may warrant consideration of additional therapeutic strategies. In recent years, M-TEER have emerged as a major therapeutic advance for severe secondary MR.^24,25^ However, more recent evidence has begun to suggest that selected patients with moderate secondary MR may also benefit.

In RESHAPE-HF2 (Transcatheter Valve Repair in Heart Failure with Moderate to Severe Mitral Regurgitation),^8^ which included patients with both moderate and severe secondary MR, M-TEER was associated with reductions in HFHs and improvements in quality of life, despite lower mean EROA values compared with the COAPT (Transcatheter Mitral-Valve Repair in Patients with Heart Failure) trial.^7^ These findings suggest that the benefit of intervention may not be limited to the most extreme MR phenotypes. Consistently, data from the EXPANDed registry demonstrated sustained MR reduction and functional improvement in patients with moderate secondary MR treated with M-TEER.^11^ However, this observational dataset remains limited by the lack of untreated control groups and by incomplete information on long-term outcomes under contemporary GDMT. Additionally, a recent systematic review of 26M-TEER registries including 18 925 patients with secondary MR showed that moderate MR represented 0%–32.6% of patients, indicating that patients with moderate secondary MR are also represented in real-world M-TEER cohorts and highlighting variability in MR grading.^26^ Therefore, although the existing evidence is provocative, definitive conclusions regarding the role of M-TEER in moderate secondary MR cannot yet be drawn. Randomized trials demonstrating improved prognosis after treatment of moderate secondary MR are required before secondary MR can be elevated from a biomarker with association to a causative influence.

Several limitations of this study should be acknowledged. Mitral regurgitation severity was assessed by TTE as part of routine clinical care, and quantitative parameters were not available in all patients, requiring an integrated qualitative and semiquantitative assessment by experienced cardiologists. Although this reflects real-world practice, it may introduce measurement variability. However, studies were consistently performed by the same operators within an expert tertiary-care echocardiography laboratory.^6^ In addition, the analysis was restricted to patients surviving to the 12 month echocardiographic reassessment, introducing a potential survivorship bias. Multiple TTEs were not performed at baseline or 12 months to assess the short-term stability of MR grading. Finally, the historical nature of the cohort and the long inclusion period resulted in heterogeneity in GDMT exposure and relatively low use of ARNI and SGLT2 inhibitors, although treatments were optimized according to contemporary standards at each time point.

In summary, in this large-scale cohort of ambulatory patients with mostly HFrEF who underwent serial TTE at baseline and 12 months after GDMT optimization, and were followed for an additional median of 6.9 years, secondary MR severity after GDMT optimization emerged as a strong and independent determinant of prognosis. Both moderate and severe secondary MR at follow-up were associated with significantly higher subsequent risks of all-cause mortality, first and recurrent HFHs, and the composite endpoint of death or HFH compared with patients with no or mild MR after GDMT. Notably, among patients with moderate secondary MR at 12 months, outcomes were similarly poor regardless of baseline MR severity, underscoring that residual regurgitation severity after treatment optimization—rather than initial MR grade—drives long-term risk. These findings highlight the clinical value of systematic echocardiographic reassessment of secondary MR following GDMT optimization, providing essential prognostic information that may improve long-term risk stratification and guide management strategies in HFrEF. Lastly, randomized trials of TEER to correct moderate secondary MR (currently in the planning stages) are necessary to determine whether moderate secondary MR is merely an established biomarker of a worse prognosis in HF, or if its presence (like severe secondary MR) infers a causative role in worsening clinical outcomes of patients with HF.

## Supplementary Material

xvag256_Supplementary_Data

## Declarations

### Data Availability

No data were generated or analysed for this manuscript.

### Pre-registered Clinical Trial Number

None supplied.
