# Supplementary material for: Prognostic impact of moderate secondary mitral regurgitation after optimized medical therapy in heart failure
Source: ESC Heart Fail. 2026 Oct 17;13(5):xvag256. doi: 10.1093/eschf/xvag256 (PMC13695058; doi:10.1093/eschf/xvag256)
Supplement: Supplementary file 1 [file xvag256_supplementary_data.pdf]

SUPPLEMENTARY MATERIAL

Prognostic Impact of Moderate Secondary Mitral Regurgitation After Optimized Medical Therapy in Heart Failure

Supplementary Figure 1. Study flowchart

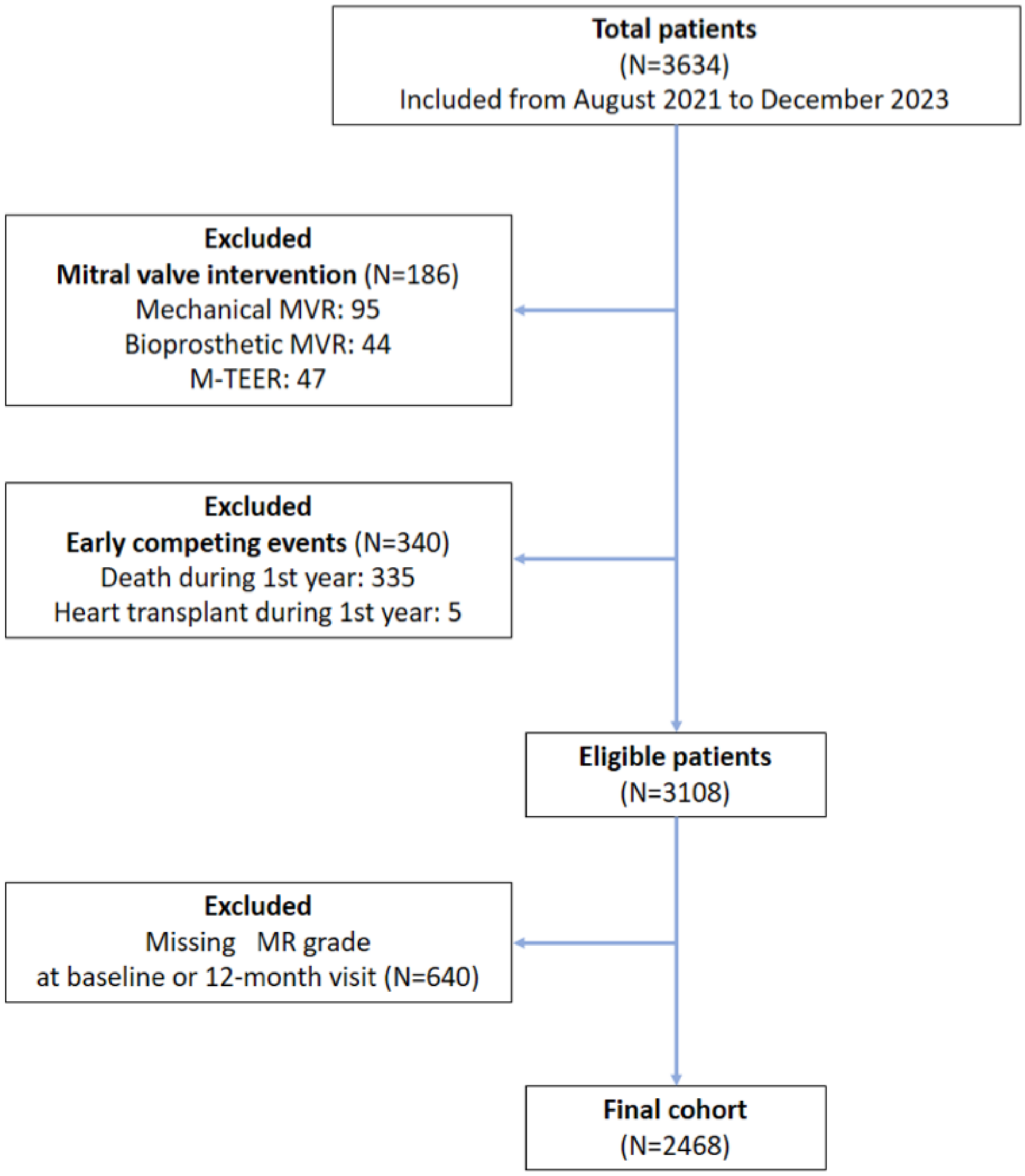

Alt Text. Flow diagram showing patient selection from 3,634 patients to a final cohort of 2,468 after exclusions. M-TEER, mitral transcatheter edge-to-edge repair; MVR, mitral valve replacement; MR, mitral regurgitation.

Supplementary Figure 2. Evolution of mitral regurgitation severity during follow-up

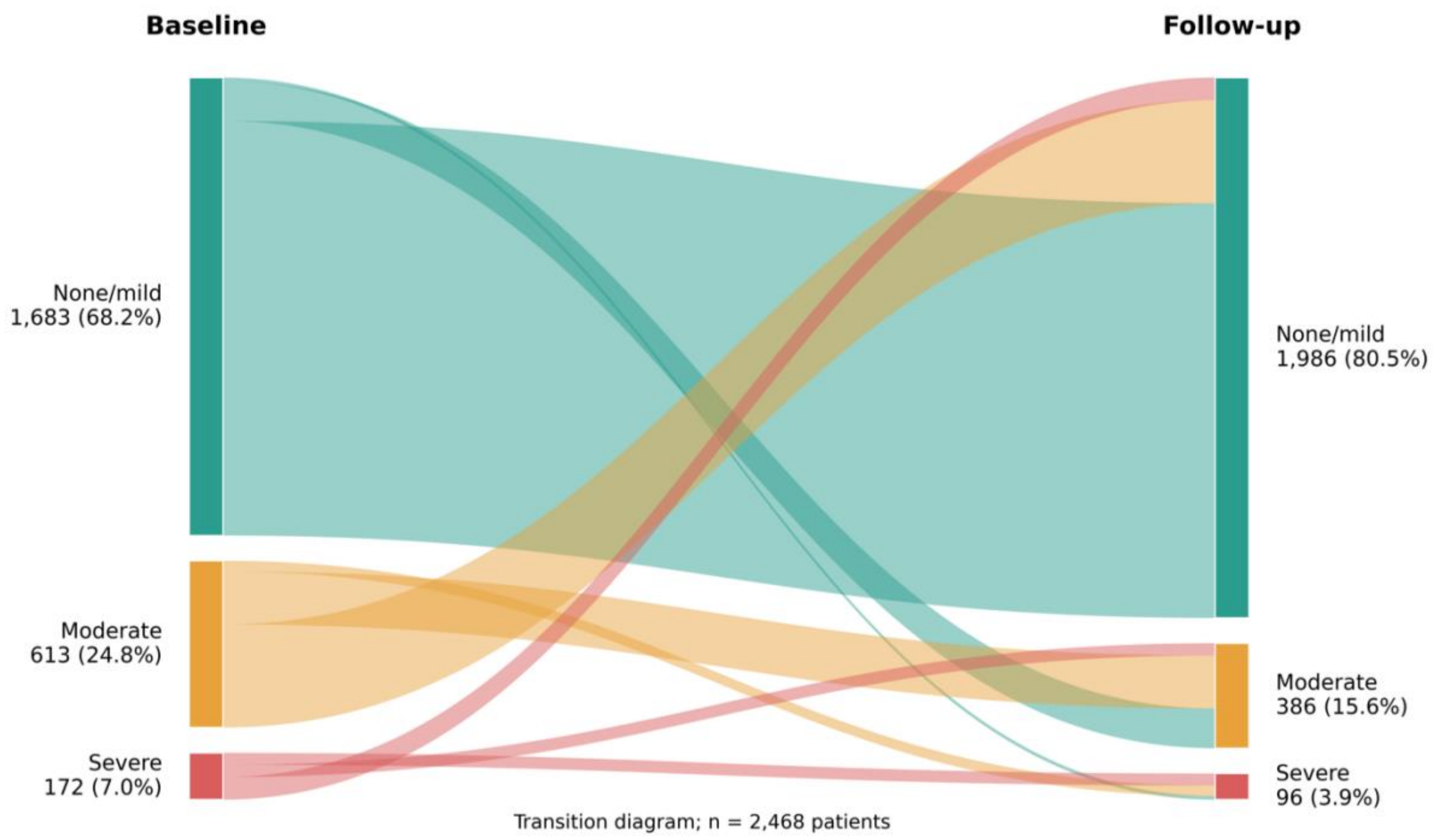

Alt text: Transition diagram showing changes in secondary MR severity from baseline to 12 months.

Supplementary Figure 3. Baseline-to–12-month secondary MR transitions in patients with moderate secondary MR at follow-up

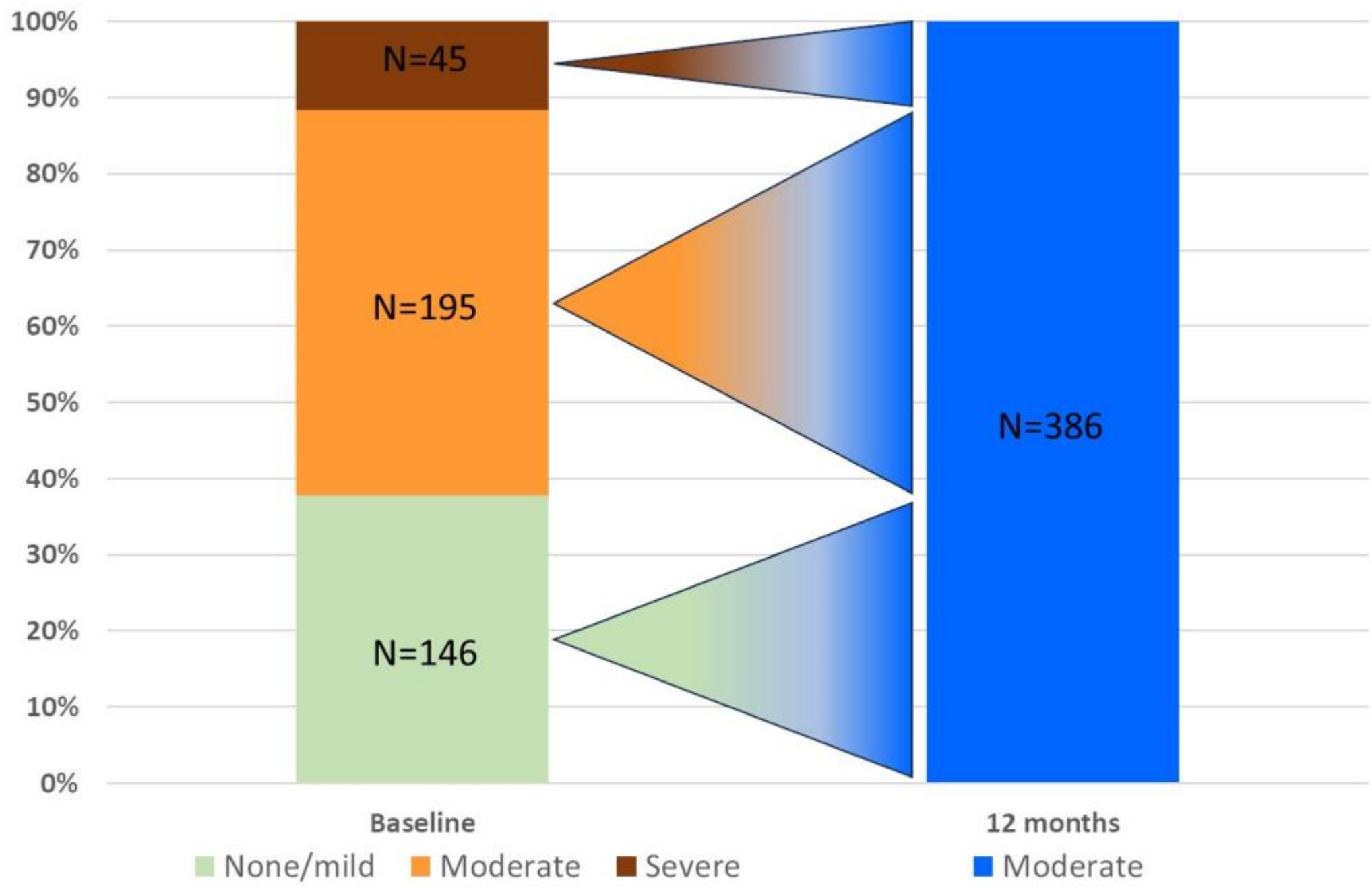

Alt text: Bar chart showing HF hospitalization rates according to baseline MR severity. Most patients had none/mild or moderate MR at baseline.

Supplementary Figure 4. Prognosis of patients with moderate secondary MR at 12 months according to baseline MR severity.

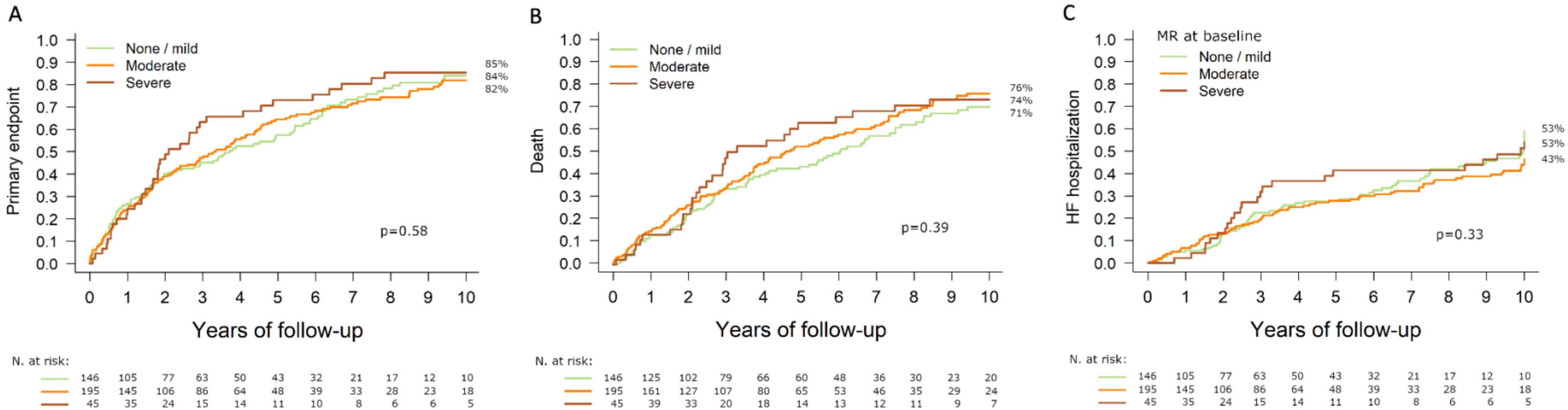

Alt text. Panel A - Cumulative incidence (Kaplan–Meier estimate) of the primary composite endpoint (all-cause death or HF hospitalization). Panel B - Cumulative incidence (Kaplan–Meier estimate) of all-cause death. Panel C - Cumulative incidence function of HF hospitalization (Fine–Gray model).

Supplementary Figure 5. Recurrent heart failure hospitalizations in patients with moderate secondary MR at 12 months stratified by baseline MR severity

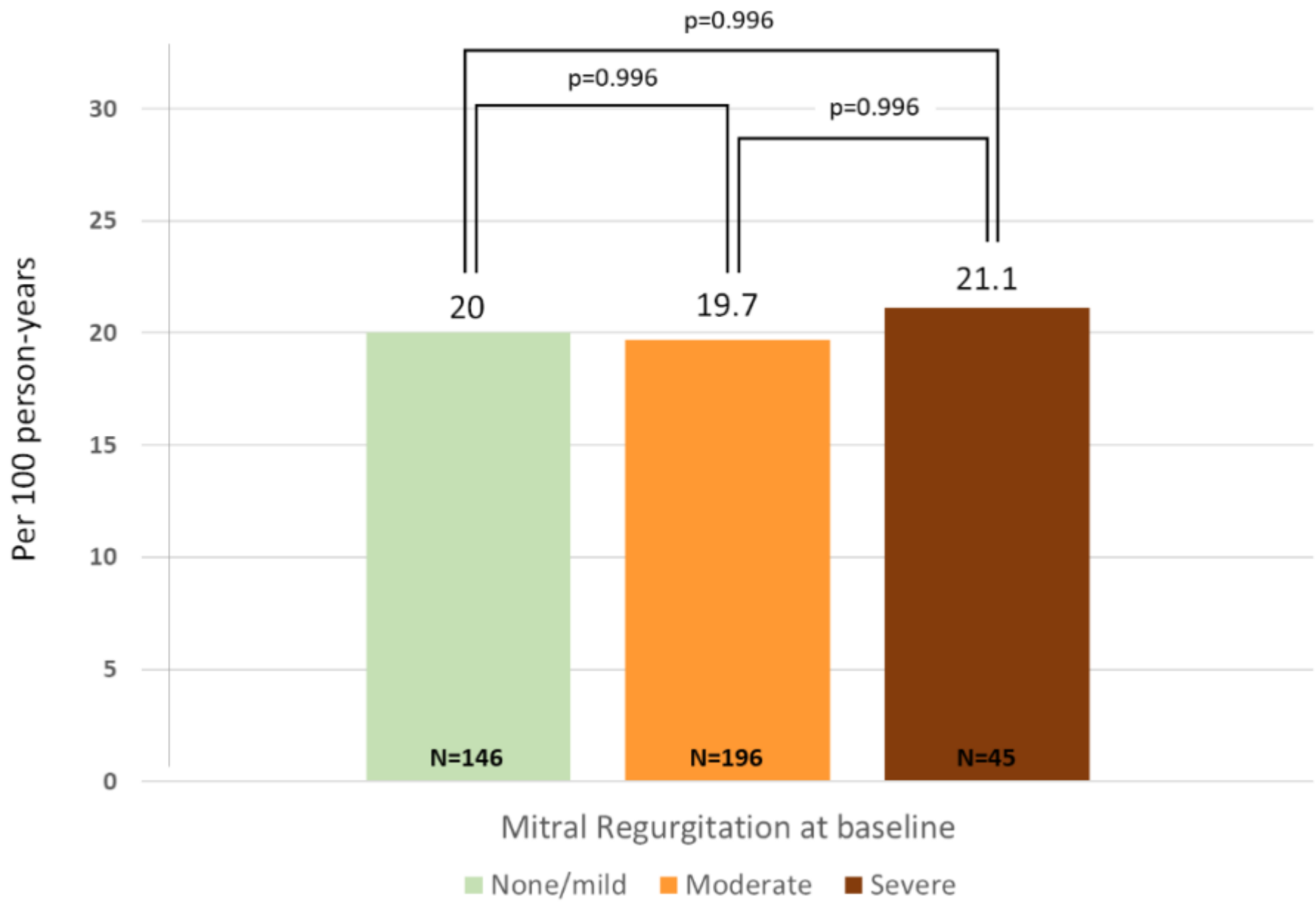

Alt text: Results per 100 person-years. P-values Holm-adjusted.

Supplementary Table 1. Comparison of baseline variables between included patients and those with missing MR assessment

Variable	Excluded (n=640)	Included (n=2468)	p value
Age (years)	65.1 ± 14.1	66.2 ± 12.5	0.068
Male sex, n (%)	453 (70.8%)	1743 (70.6%)	0.977
Heart failure characteristics			
LVEF (%)	39.2 ± 16.2	36.1 ± 13.6	<0.001
Heart failure duration (months)	12 [2–48]	6 [1–36]	<0.001
NYHA class, n (%)			
Class 1	63 (9.8%)	215 (8.7%)	<0.001
Class 2	385 (60.2%)	1758 (71.3%)	
Class 3	182 (28.4%)	485 (19.7%)	
Class 4	10 (1.6%)	7 (0.3%)	
Atrial fibrillation or flutter, n (%)	123 (19.2%)	525 (21.3%)	0.278
Ischemic etiology, n (%)	270 (42.2%)	1100 (44.6%)	0.3
Comorbidities			
Diabetes mellitus, n (%)	275 (43.0%)	993 (40.2%)	0.227
Hypercholesterolemia, n (%)	333 (52.0%)	1369 (55.5%)	0.13
NT-proBNP (pg/mL)	1398 [503–3134]	1460 [613–3345]	0.276
Hemoglobin (g/dL)	13.1 ± 1.9	13.2 ± 1.8	0.768
Treatment at 1-year			
ACEi/ARB, n (%)	383 (59.8%)	1758 (71.2%)	<0.001
BB, n (%)	462 (72.2%)	2248 (91.1%)	<0.001
ARNI, n (%)	68 (10.6%)	573 (23.2%)	<0.001
MRA, n (%)	282 (44.1%)	1584 (64.2%)	<0.001
Loop diuretic, n (%)	447 (69.8%)	2058 (83.4%)	<0.001
SGLT2 inhibitors, n (%)	64 (10.0%)	537 (21.8%)	<0.001
Ivabradine, n (%)	70 (10.9%)	413 (16.7%)	<0.001
Digoxin, n (%)	117 (18.3%)	545 (22.1%)	0.041

ICD, n (%)	43 (6.7%)	291 (11.8%)	<0.001
CRT, n (%)	11 (1.7%)	163 (6.6%)	<0.001
Composite primary endpoint, n (%)	376 (58.8%)	1240 (50.2%)	<0.001

Values are mean ± standard deviation, n (%), or median [interquartile range].

ACEi, angiotensin-converting enzyme inhibitor; AF, atrial fibrillation; ARB, angiotensin II receptor blocker; ARNI, angiotensin receptor–neprilysin inhibitor; BB, beta-blocker; COPD, chronic obstructive pulmonary disease; CRT, cardiac resynchronization therapy; eGFR, estimated glomerular filtration rate; ICD, implantable cardioverter-defibrillator; MRA, mineralocorticoid receptor antagonist; NT-proBNP, N-terminal pro B-type natriuretic peptide; NYHA, New York Heart Association; SGLT2i, sodium–glucose cotransporter 2 inhibitor.

Supplementary Table 2. Multivariable Fine–Gray model for first HF hospitalization

**Variable**	**Adjusted sHR (95% CI)**	**p value**
Secondary MR group 2 vs 1	1.79 (1.47–2.17)	<0.001
Secondary MR group 3 vs 1	1.48 (1.05–2.10)	0.025
Age (per year)	1.02 (1.02–1.03)	<0.001
Female sex	0.94 (0.78–1.12)	0.48
Ischemic etiology	1.16 (0.98–1.38)	0.078
LVEF at 12 months (per %)	0.98 (0.98–0.99)	<0.001
HF duration (log)	1.10 (1.05–1.14)	<0.001
Diabetes mellitus	1.39 (1.19–1.63)	<0.001

MR group 1 corresponds to mild or no MR, group 2 to moderate MR, and group 3 to severe MR. sHR, subdistribution hazard ratio; HF, Heart failure; LVEF, Left ventricular ejection fraction; IRR, Incidence rate ratio. MR, mitral regurgitation.

Supplementary table 3. Multivariable predictors of all (first and recurrent) HF hospitalizations

Variable	Adjusted IRR (95% CI)	p value
Secondary MR group 1 (None/mild)	Reference	—
Secondary MR group 2 (moderate)	2.17 (1.81–2.60)	<0.001
Secondary MR group 3 (severe)	2.63 (1.93–3.58)	<0.001
Age (per year)	1.04 (1.03–1.05)	<0.001
Female sex	1.08 (0.92–1.27)	0.357
Diabetes mellitus	1.72 (1.49–1.98)	<0.001
LVEF at 12 months (per %)	0.98 (0.97–0.98)	<0.001
HF duration (log)	1.15 (1.11–1.20)	<0.001

MR group 1 corresponds to mild or no MR, group 2 to moderate MR, and group 3 to severe MR. HF, Heart failure; IRR, Incidence rate ratio; LVEF, Left ventricular ejection fraction; MR, Mitral regurgitation.

Supplementary Table 4. Multivariable predictors of time to first death or heart failure hospitalization in patients with moderate secondary MR at 12 months according to baseline MR severity

Variable	Multivariable HR (95% CI)	p value
Baseline MR severity		
None/mild	Reference	—
Moderate	1.010 (0.773–1.320)	0.939
Severe	1.159 (0.790–1.698)	0.451
Age (per 5 years)	1.226 (1.149–1.308)	<0.001
Female sex	0.817 (0.625–1.068)	0.140
Ischemic etiology	1.134 (0.870–1.478)	0.351
LVEF at 12 months (per 5%)	0.940 (0.896–0.987)	0.012
HF duration (log)	1.013 (0.947–1.084)	0.702
Diabetes mellitus	1.393 (1.086–1.786)	0.009

HF, Heart failure; IRR, Incidence rate ratio; LVEF, Left ventricular ejection fraction; MR, Mitral regurgitation.

Supplementary Table 5. Multivariable predictors of all-cause mortality in patients with moderate secondary MR at 12 months stratified by baseline MR severity

Variable	Multivariable HR (95% CI)	p value
Baseline MR severity		
Mild/none	Reference	—
Moderate	1.210 (0.905–1.617)	0.198
Severe	1.272 (0.832–1.944)	0.266
Age (per 5 years)	1.306 (1.215–1.403)	<0.001
Female sex	0.712 (0.531–0.953)	0.023
Ischemic etiology	0.894 (0.670–1.194)	0.448
LVEF at 12 months (per 5 %)	0.943 (0.896–0.993)	0.025
HF duration (log)	1.044 (0.970–1.125)	0.250
Diabetes mellitus	1.499 (1.147–1.959)	0.003

HF, Heart failure; IRR, Incidence rate ratio; LVEF, Left ventricular ejection fraction; MR, Mitral regurgitation.
